# Maximal Resection of Tumors Encasing the Internal Carotid Artery and Hindering Internal Carotid Artery Expansion Followed by Revascularization Surgery: A Series of Nine Cases at a Single Tertiary Center

**DOI:** 10.3389/fsurg.2022.808446

**Published:** 2022-02-17

**Authors:** Yuming Sun, Long Wang, Xiangen Shi, Fangjun Liu

**Affiliations:** ^1^Department of Neurosurgery, Fuxing Hospital, Capital Medical University, Beijing, China; ^2^Department of Neurosurgery, SanBo Brain Hospital, Capital Medical University, Beijing, China

**Keywords:** middle cranial fossa tumors, cerebral revascularization, extracranial-intracranial bypass, internal maxillary artery, craniopharyngioma, meningioma

## Abstract

**Purpose:**

Cerebral reconstruction appears to play a diminished role in managing complex skull base tumors involving vital neurovascular structures.

**Materials and Methods:**

Patients with recurrent or progressive middle cranial fossa tumors treated by radical resection followed by extracranial-to-intracranial (EC-IC) bypass from 2014 to 2019 were included. Balloon test occlusion (BTO) was performed preoperatively.

**Results:**

Overall, 9 patients (5 males, 4 females; mean age, 29.9 years) were enrolled. The lesions arose from the parasellar region (3), cavernous sinus (3), petroclival region (2), or orbital apex (1), and all encased the cavernous/petrous portion of the internal carotid artery. Before tumor resection, internal maxillary artery (IMA) bypass was performed for 7 patients, cervical EC-IC bypass was performed for 1 patient, and interposed superficial temporal artery (STA) bypass was performed for 1 patient. BTO failed in 8 patients and was tolerated by one patient. Intraoperative blood flow of the interposed graft was 79.7 ± 37.86 ml/min after IMA bypass, 190.6 ml/min following cervical EC-IC bypass and 75 ml/min after interposed STA bypass. All bypasses were patent on intraoperative indocyanine green angiography. Radical tumor resection was achieved in 5 patients (55.6%), and patency was confirmed postoperatively in 88.8% (8/9) of bypasses. Six patients showed favorable outcomes at discharge. At the 2-year follow-up, 7 patients (77.8%) had favorable outcomes (Karnofsky Performance Scale score>80). At the 1.5-year follow-up, one patient had died due to infarction; at the 3-year follow-up, another patient had developed tumor recurrence despite being asymptomatic.

**Conclusion:**

Cerebral bypass remains a vital tool for managing select middle cranial fossa tumors that invade or erode the surrounding neurovasculature or hinder carotid artery expansion and are difficult to resect.

## Introduction

Recently, the majority of reports (1–3) on middle cranial fossa tumors have described the increased use of the endoscopic endonasal approach (EEA), but this treatment is still unsatisfactory for patients with tumor encasement or invasion of the cavernous/petrous portions of the internal carotid artery (ICA) (4, 5). In this series, 9 patients, accounting for 1.4% of 621 patients surgically treated for middle fossa floor or sellar region tumors, underwent maximal resection of middle cranial fossa tumors followed by bypass surgery. After the carotid artery bypass, the complex middle cranial fossa tumors encasing/invading the ICA were maximally removed, thereby minimizing surgical difficulty.

## Materials and Methods

### Preoperative Preparation

We retrospectively reviewed 9 patients with recurrent or progressive middle cranial fossa tumors encasing or invading the ICA and were treated from 2014 to 2019. These patients were subjected to aggressive tumor resection with resection of the ICA, followed by revascularization *via* internal maxillary artery (IMA) bypass, extracranial-to-intracranial (EC-IC) bypass with the great saphenous vein (GSV) or superficial temporal artery (STA) interposition bypass at our institute. Preoperative evaluation was performed for all patients by magnetic resonance imaging (MRI), computed tomography angiography (CTA), digital subtraction angiography, and CT perfusion imaging. Positron emission tomography (PET) was performed for 5 patients to define the cerebrovascular reserve with hemodynamic effects. Balloon test occlusion (BTO) was performed for all 9 patients to assess cross-collateral flow patency through the circle of Willis. Follow-up examinations were conducted in an outpatient clinic or *via* short message service, mobile call, and WeChat interviews if an in-person visit was not possible. All patients underwent follow-up CT/MRI at 3 and 6 months and then yearly thereafter for postoperative radiographic evaluation, and recurrence and death related to surgery at the end of the follow-up period were recorded. The Karnofsky Performance Scale (KPS) was used to evaluate clinical outcomes; a KPS score of more than 80 was considered a good outcome; 60–70, fair; and < 50, poor (see Table 1).

**Table 1:** Demographic data of 9 patients of skull base tumors treated with adjunctive bypass surgery.

**Case**	**Sex/** **age** **(yrs)**	**Diagnosis**	**NO**	**Side/degree of CS involvement and ICA encasement**	**Approches**	**Bypass**	**FV**	**Extent of tumor resection**	**Graft patency**	**Complications**	**KPS scale** **Pre-/post-surgery**
1	M/14	Recurrent Craniopharyngioma +FDICA	2	R/II (C5-C7)	Frontotemporal (OZO)	IMA-RA-M2	50	RR	Yes	None	>80/>80
2	F/12	Recurrent Craniopharyngioma +FDICA	2	R/II (C5-C6)	Frontotempora (OZO)	IMA-RA-M2	91	RR	Yes	None	>80/>80
3	F/50	Recurrent hemangiopericytoma	3	R/IV (C3-C6)	Frontotemporal (OZO)	IMA-RA-M2	70	STR	Yes	None	>80/>80
4	M/28	Recurrent meningioma	4	R/III (C4-C7)	Frontotemporal (OZO)	IMA-RA-M2	100	GTR	No	Cerebrospinal fluid leakage, secondary hydrocephalus, uneventful through V-P shunt	70/60
5	F/47	Recurrent meningioma	3	R/IV (C4-C7)	Frontotemporal (OZO)	IMA-RA-M2	80	GTR	Yes	Delayed intracranial hematoma and died onthe posteroperstive 17th day	60/40
6	M/35	Recurrent chordoma	4	R/IV (C5-C6)	Frontotemporal (OZO)	ECA-SV-M2	120	STR	Yes	Cerebral infarction and decompressive craniectomy, died on the sixth month	50/30
7	F/30	Recurrent adenoid cystic carcinoma	2	L/IV (C3-C6)	Frontotemporal (OZO)	IMA-RA-M2	47	GTR	Yes	None	>80/>80
8	M/46	Meningioma	1	L/IV (C3-C6)	Frontotemporal (OZO)	IMA-RA-M2	45	GTR	Yes	None	>80/>80
9	M/35	Primary chordoma	1	R/IV (C3-C6)	Frontotemporal (OZO)	STA interposition	35	GTR	No	Died fromhemishperic infarction at 1.5 years follow-up after surgery	>80/20

*M, male; F, femal; R, right; L, left; B, bilateral; NO, number of operations; FDICA, Fusiform dilation of internal carotid artery; ICA, the involed internal carotid artery; RA, radial artery; STA, superficial temporal artery; ECA, external carotid artery; M2, M2 segment of middle cerebral artery; FV, flow volume of graft (ml/min); OZO, orbitozygomatic osteotomy; RR, radical resection; GTR, gross total resection; STR, subtotal resection*.

### Patient Selection

Surgery was indicated in 6 patients due to postradiotherapy signs of recurrent or malignant tumors encasing the cavernous or petrous portion of the ICA, in which preoperative angiography indicated compromise of the carotid lumen, suggestive of direct invasion of the vessel wall with a high risk of intraoperative vessel injury. The decision-making process and management principles for carotid artery resection were replaced by those for grafts until all the factors of the individual patients were considered, including a previous history of surgical retreatment and radiation therapy and the feasibility of aggressive surgical resection for young patients with a long life expectancy. Two patients with fusiform ICA dilation were treated with IMA bypass subsequent to resection of the craniopharyngioma of the medial wall of the cavernous sinus due to recurrent tumors in this region invading the carotid artery and the ability to expose the tumor through resection of the fusiform ICA dilation after IMA bypass. STA interposition bypass was performed between the proximal and distal cut ends of the supraclinoid ICA in 1 patient following an accidental tear of the paraclinoid ICA during tumor dissection from the invaded ICA; vascular reconstruction was performed without a preoperative plan.

### Surgical Procedures

Routine frontotemporal craniotomy was performed by cutting along the zygomatic arch to facilitate downward retraction of the temporal muscle; this procedure was adapted for the floor of the middle cranial fossa for adequate exposure of the parasellar region, middle cranial fossa and petrous apex if required. The details of the operative procedures are similar to those previously published in the literature (6–8). Anterior clinoidectomy was performed to expose the clinoid ICA, optic canal and orbital apex, and then the fusiform dilation was opened to decompress the optic nerve. For benign tumors, piecemeal tumor removal with identification of its cranial nerve-free segments was performed after the tumor was meticulously dissected from the cranial nerves through the cavernous sinus. For malignant tumors, the tumor was debulked using an ultrasound dissector or powerful suction after the cranial nerves were freed from the tumor mass. Oculomotor preservation was used as an intraoperative marker to evaluate cranial nerve integrity through the cavernous sinus; the oculomotor nerve was preserved, sacrificed, or remained unidentified during surgery. Prophylactic IM EC-IC bypass with radial artery (RA) grafting was performed for 7 patients, cervical EC-IC bypass with the GSV was performed for 1 patient, and STA interposition bypass between the proximal and distal cut ends of the supraclinoid ICA tear was performed for 1 patient. The blood flow rate measured intraoperatively by ultrasound (BK Medical) ranged from 47–120 ml/min (mean, 79.7 ± 37.86 ml/min) in patients who underwent IM EC-IC bypass, while it was 190.6 ml/min in the patient who underwent EC-IC bypass with the GSV and 75 ml/min in the patient who underwent STA interposition bypass. Intraoperative angiography with indocyanine green (ICG) confirmed graft patency in all 9 patients. In 2 patients, the confluent sellar-sphenoid cavity opened postoperatively and was packed with a small plug of muscle and pieces of fascia, and in 4 patients, the opened confluent paranasal sinus-intersellar turcica-middle cranial fossa was repaired with 7–0 nylon watertight microsuturing of the superficial fascia of the temporal muscle around the dural edge of the defective dural cavity. Before occlusion, patients received IV heparin (20–30 units/kilogram) to prevent postprocedural thrombosis of the graft vessel; immediate salvage strategies for IMA bypass have previously been published (9, 10). Revascularization was performed after barbiturate protection and the provision of mild hypothermia. Intraoperative monitoring by somatosensory-evoked potentials and electroencephalography was performed. During arterial occlusion, the systolic blood pressure was increased by 25–30% over baseline.

## Results

### General Patient Baseline Characteristics and Tumor Features

A total of 9 patients were assessed, including 5 males and 4 females, ranging in age from 12 to 50 years (mean, 29.9 years). Recurrent tumors were observed in 8 patients, and primary tumors were observed in 1 patient. The surgical outcomes and postoperative courses are detailed in Table 1. The tumors originated from the parasellar region in 3 patients; the cavernous sinus, 3 patients; the petroclival region, 2 patients; and the orbital apex, 1 patient. Five patients had regularly shaped tumors with a maximal diameter between 4.5 and 11.3 cm (mean diameter, 6.3 cm). Irregularly shaped tumors were observed in the remaining 4 patients. Extension from the petroclival area to the middle fossa and clivus was observed in 2 patients; extension from the orbital apex to the interstellar region and cavernous sinus, 1 patient; and extension from the parasellar region to the maxillary sinus and nasal cavity, 1 patient. In addition, tumor encasement of the supraclinoid ICA with an unidentified plane between the ICA and tumor was observed in all 9 patients. Angiography revealed tumors encasing or invading the cavernous ICA in 6 patients and the petrous ICA in 3 patients.

### Bypass Surgery and Tumor Resection

**Figure 1:**
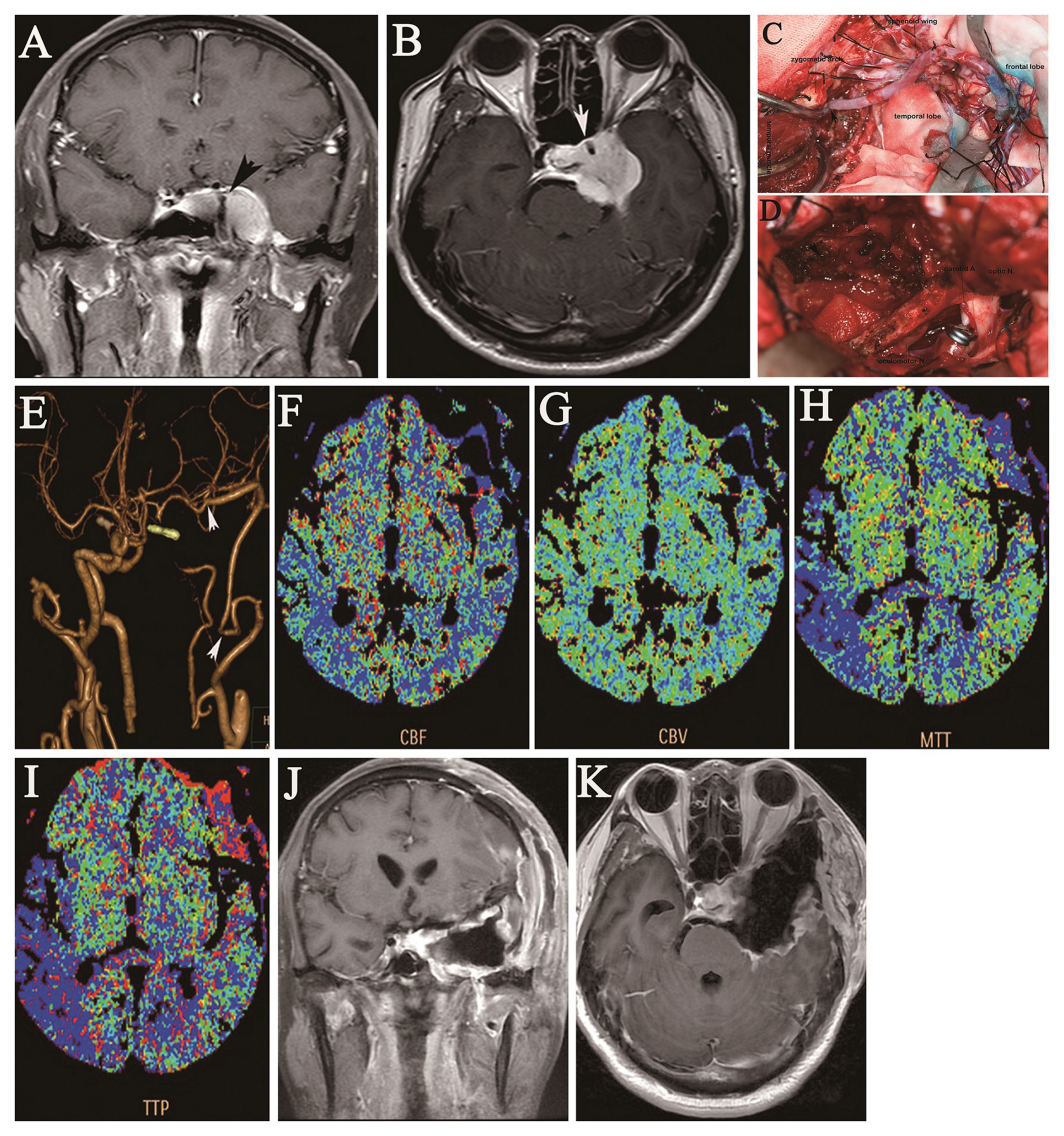
A 46-year-old man presented with a 7-year history of double vision and left-sided facial numbness for 1 year and ptosis for half a year. Coronal **(A)** and axial **(B)** preoperative contrast-enhanced MRI shows a large tumor in the cavernous region encasing the ICA **(A)**, as indicated by the arrow. Intraoperative images show end-to-end anastomosis of the IMA to the RA and end-to-side anastomosis of the M2 segment of the MCA through a frontotemporal craniotomy with resection of the zygomatic arch **(C)**, the operative field of the cavernous region (as indicated by the arrow), and the clipped supraclinoid ICA after total tumor resection **(D)**. Postoperative CTA shows the patency of the IMA bypass with the RA graft and occlusion of the left ICA; the arrows indicate the anastomotic sites of the RA graft **(E)**. Postoperative CT perfusion imaging shows the **(F)** cerebral blood flow, **(G)** cerebral blood volume, **(H)** mean transit time, and **(I)** time-to-peak for the left cerebral hemisphere after IMA bypass, which is consistent with symmetry in the hemodynamic capacity of the right cerebral hemisphere. Coronal **(J)** and axial **(K)** contrast-enhanced MRI at the 2-year follow-up shows no tumor growth in the cavernous region.

**Figure 2:**
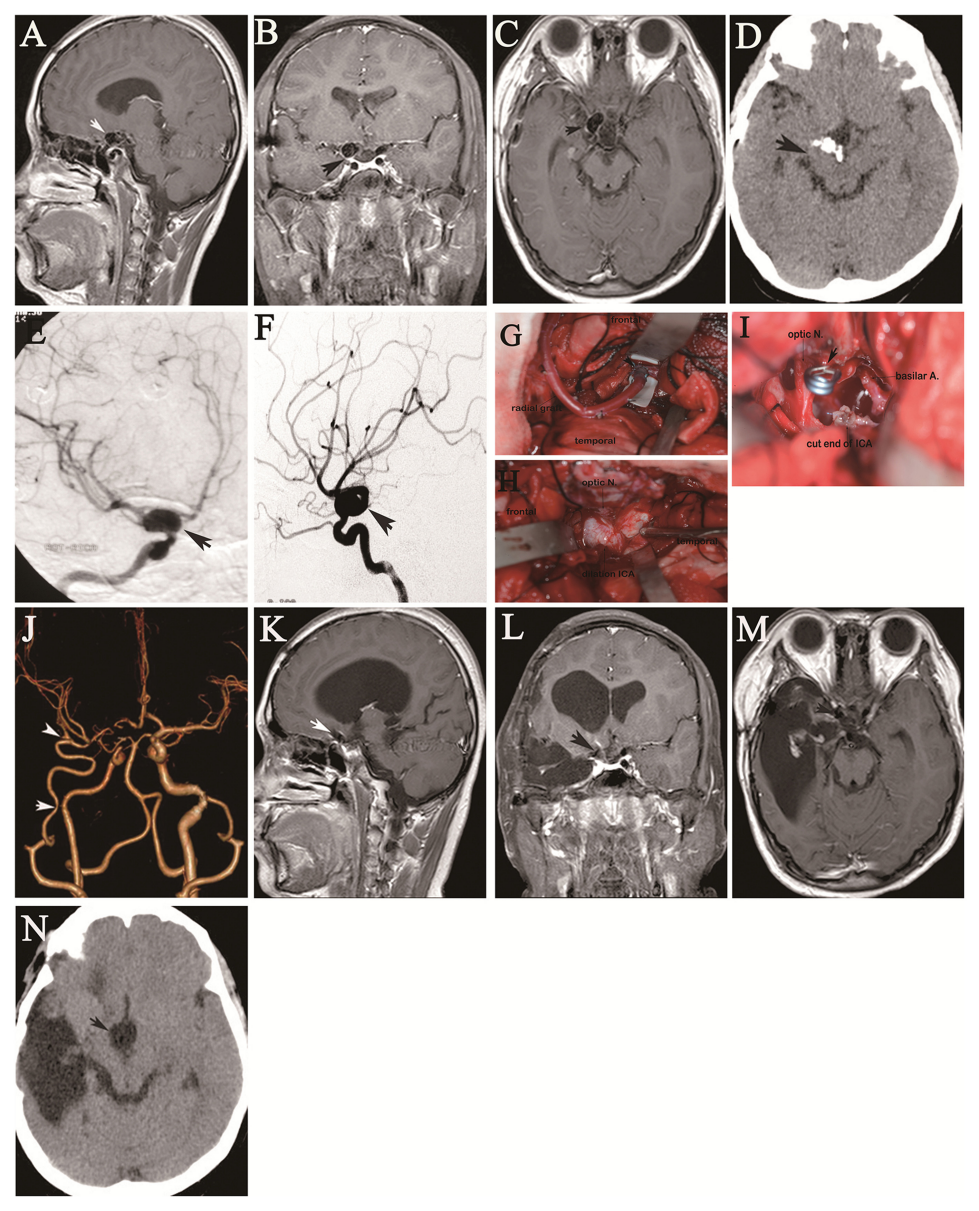
A 13-year-old girl was referred to our hospital with a 3-month history of severe headache and vomiting. Her neurological function was intact on admission. More than 5 years (September 2009) before she was referred to our department, she had undergone gross total resection of a craniopharyngioma at another institution. Preoperative sagittal **(A)**, coronal **(B)**, and axial **(C)** preoperative contrast-enhanced MRI shows the tumor in the intrasellar region complicated by fusiform ICA dilation (arrow). Preoperative axial CT **(D)** shows irregular tumor calcification. Preoperative right oblique **(E)** and lateral **(F)** DSA confirms dilation of the supraclinoid ICA with a maximum diameter of 21 mm (arrows). Intraoperative images show end-to-side anastomosis of the RA graft to the proximal M2 segment of the MCA *via* an opened Sylvian fissure through a frontotemporal craniotomy with resection of the zygomatic arch (arrow indicates the anastomotic site) **(G)**. Image shows fusiform dilation lesion of the right supraclinoid ICA through exposure of the suprasellar region (arrow) **(H)**. Image shows resection of the fusiform lesion, followed by clipping of the proximal and distal segments of the fusiform ICA dilation, and then resection of the tumor in the sellar region through the space left by resection of the fusiform ICA dilation (arrow) **(I)**. Postoperative CT angiography showed patency of the IMA to the MCA and supraclinoid ICA sacrifice (the arrows indicate the anastomotic sites) **(J)**. Follow-up sagittal **(K)**, coronal **(L)**, and axial **(M)** contrast-enhanced MRI at 3.5 years shows no tumor growth in the sellar region (arrow) and a right temporal pole defect related to evacuation of the postoperative hematoma. Axial CT demonstrates disappearance of tumor calcification **(N)**.

**Figure 3:**
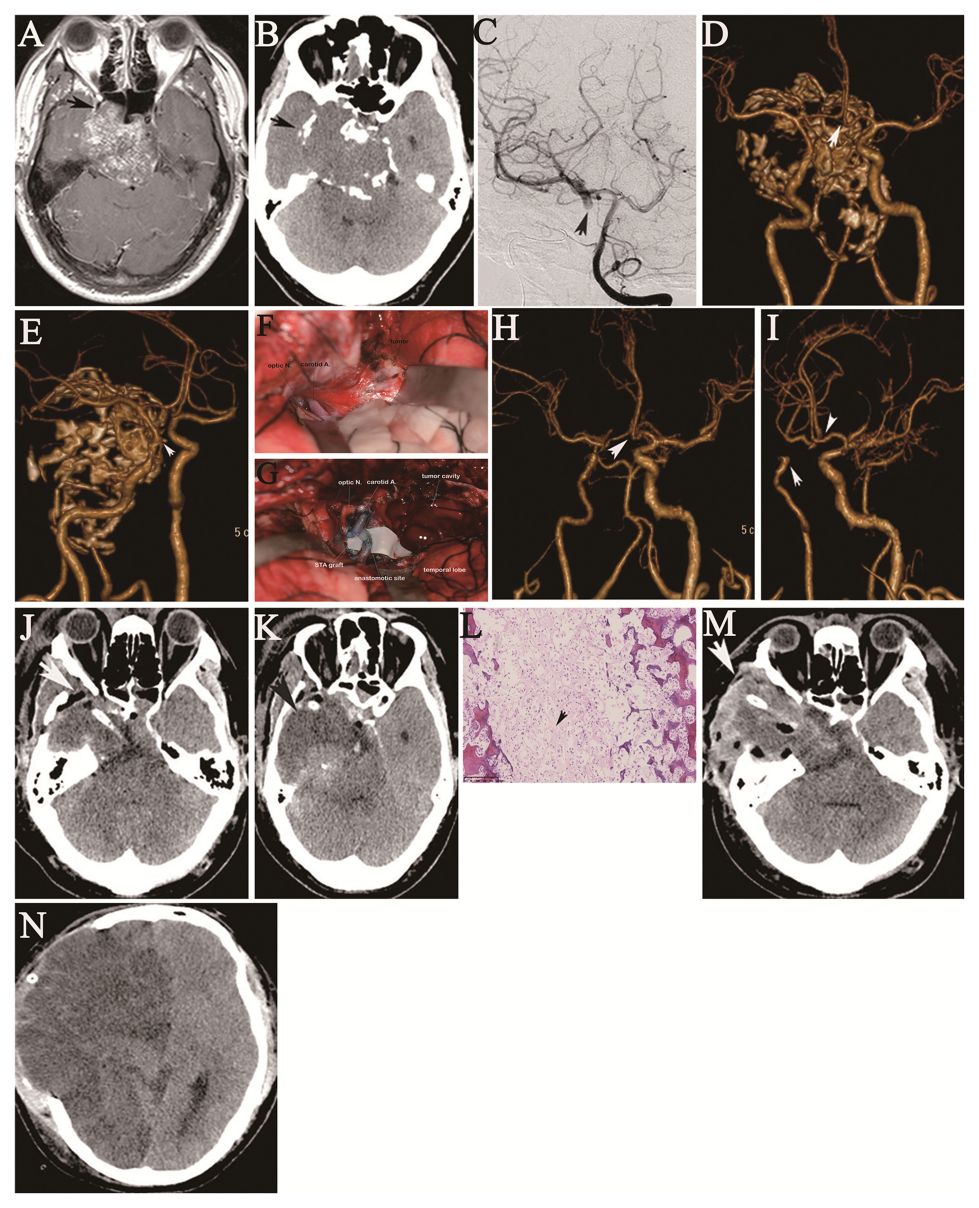
A 36-year-old man had deteriorating double vision for two years and a 1-week history of right facial numbness, resulting in the inability to work as a truck driver. Axial preoperative contrast-enhanced MRI shows a large tumor in the sphenopetroclival area encasing the cavernous ICA **(A)**, and axial CT reveals the tumor around fragments of destroyed bone **(B)**, as indicated by the arrows. Angiography of the left vertebral artery shows full cross-filling of the right PComA to the right ICA after balloon occlusion of the right ICA, as indicated by the arrow **(C)**. Anterior **(D)** and oblique **(E)** CTA shows the tumor invading the supraclinoid ICA (arrow). Intraoperative images show the exposed tumor in the anterior area of the middle cranial fossa and parasellar region through a frontotemporal craniotomy **(F)** and the interposed STA graft between the proximal and distal cut ends of the ICA caused by a supraclinoid ICA tear during resection of the tumor in the sphenopetroclival area **(G)**. Postoperative anteroposterior **(H)** and oblique **(I)** CTA shows the cutoff filling of the right ICA to the right MCA (arrow), with cross-filling of the left ACA to the right ACA (arrow). On the first day after surgery, two axial CT sections **(J,K)** show gross resection of the tumor in the sphenopetroclival area with normal brain features (arrows). Photomicroscopic image of a cranial chordoma shows characteristic physaliphorous cells (arrow) in the abundant cartilaginous matrix. Hematoxylin and eosin staining; original magnification x100 **(L)**. Four days after surgery, two axial CT sections **(M,N)** show a malignant MCA territory infarction with large bone flap decompression.

All 9 patients underwent prophylactic EC-IC bypass and interposition bypass to ensure anterior circulation territory perfusion and perforator flow, including IMA bypass with RA grafting in 7 patients (Figures 1, 2), EC-IC bypass with the GSV in 1 patient (external carotid artery-middle cerebral artery (ECA-MCA) bypass) and STA interposition bypass in 1 patient (Figure 3). Total tumor resection was achieved in 6 patients (66.7%); gross total resection, 2 patients (22.2%); and subtotal resection, 1 patient (11.1%). The oculomotor nerve was preserved in 4 patients; sacrificed in 4 patients; and unidentified in 1 patient.

### Postoperative Outcomes and Follow-Up

One death occurred 17 days after surgery due to a delayed cerebral hematoma, possibly related to therapeutic anticoagulant treatment. Cerebrospinal fluid (CSF) leakage occurred in 1 patient; surgical dural defect repair was performed *via* endoscopic endonasal surgery 2 months postoperatively. One patient exhibited an asymptomatic frontal opercularis infarction related to bypass occlusion during the perioperative period. Postoperative angiography confirmed bypass patency in 8 patients (88.9%); the interposed STA in the remaining patient was not patent (11.1%). Overall, favorable outcomes were obtained in 6 patients (66.7%) at discharge, and poor outcomes were observed in 2 patients (22.2%); one death (11.1%) caused by delayed cerebral hematoma occurred 17 days after surgery. Eight patients underwent clinical follow-up, with an average follow-up time of 30.5 months (range, 5–60 months). The 2-year outcomes were favorable (KPS score, >80) for 7 patients (77.8%) and poor (KPS score, 50 to 60) for 1 patient (11.1%), and 1 patient (11.1%) died from hemispheric infarction at the 1.5-year follow-up. Tumor recurrence was found in one patient who was asymptomatic at the 3-year follow-up. Malignant tumor recurrence in the orbital cavity occurred in 1 patient 1.5 years after surgery; the patient underwent surgery for the recurrent tumor.

### Case Illustration

**Figure 4:**
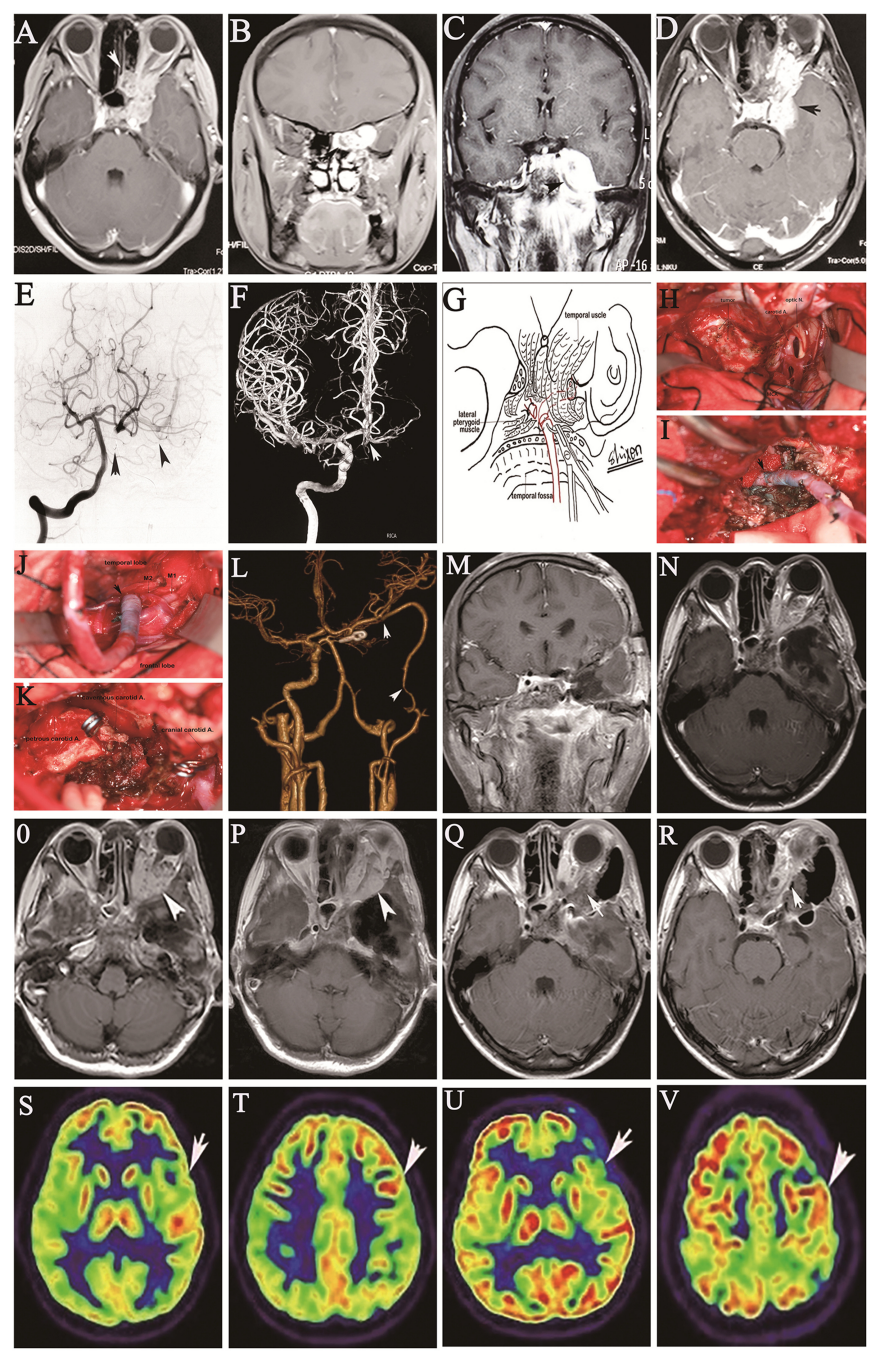
Coronal **(A)** and axial **(B)** preoperative contrast-enhanced MRI shows a left orbital apex tumor extending to the ethmoid sinus before endoscopic transnasal surgery. Three months after endoscopic transnasal surgery, coronal **(C)** and axial **(D)** MRI with contrast shows regrowth of the tumor in the left cavernous sinus, orbital cavity and apex and middle cranial fossa. Angiography shows partial cross-filling of the right PComA to the right intracranial ICA after balloon occlusion of the cervical ICA on the left vertebral angiogram; the arrow indicates a balloon in the lumen of the cervical ICA, and the arrowheads indicate the right MCA **(E)**. Rare cross-filling of the right AComA to the left ACA after balloon occlusion of the cervical ICA is shown on left ICA angiography **(F)**. Drawing of the surgical procedure shows end-to-end anastomosis of the RA grafted to the IMA in the inferior temporal fossa through a frontotemporal craniotomy with resection of the zygomatic arch **(G)**. Intraoperative images show the tumor in the cavernous and petrous regions following opening of the Sylvian fissure through a frontotemporal craniotomy with resection of the zygomatic arch **(H)**, end-to-end anastomosis of the RA grafted to the IMA in the inferior fossa **(I)**, end-to-side anastomosis of the RA grafted to the proximal M2 segment of the MCA **(J)**, and the operative field after gross total resection of the tumor with encasement of the cavernous ICA; two clips of the distal and proximal cut ends of the cavernous ICA are also visualized **(K)**. Postoperative anteroposterior CTA shows patency of the IMA bypass to the M2 segment of the MCA; the arrows indicate the anastomotic sites **(L)**. Coronal **(M)** and axial **(N)** postoperative contrast-enhanced MRI demonstrate gross resection of the cavernous sinus tumor; the arrow indicates the sacrificed ICA. The tumor invading the intraorbital cavity was residual. At the two-year follow-up, two axial contrast-enhanced MRI sections revealed stable status of tumor **(O,P)**. Two postoperative axial contrast-enhanced MRI sections **(Q,R)** show subtotal resection of the recurrent tumor in the orbital cavity (arrow). Two preoperative sections from 18-fluoro-2-deoxy-D-glucose (18F-FDG) PET/CT show the left hemisphere with normal metabolism before replacement bypass of the left ICA; the arrow indicates the MCA territory **(S,T)**. At the three-month follow-up, two 18F-FDG PET/CT sections demonstrate no significant difference in metabolism from before replacement bypass of the left ICA; the arrow indicates the replacement bypass territory **(U,V)**.

A 30-year-old female presented with a history of progressive loss of visual acuity for 3 months. MRI demonstrated a left orbital apex tumor extending from the medial ethmoid sinus to the cavernous sinus (Figures 4A,B). Transnasal biopsy revealed adenoid cystic carcinoma. She underwent endoscopic transnasal surgery at another hospital, and subtotal resection was achieved, but worsening vision, progressive diplopia, ipsilateral exophthalmos, and ophthalmodynia subsequently developed. Physical examination revealed obviously diminished visual acuity in the left eye and partial ophthalmoplegia with ipsilateral ptosis. Follow-up imaging examinations revealed tumor recurrence in the left paranasal sinus and cavernous sinus, with tumor encasement of the petrous and cavernous portions of the ICA extending forward into the orbital apex, involving some of the eye muscles and protruding down into the sphenoid sinus and medial petrous bone (Figures 4C,D). Angiography revealed partial cross-filling of the left posterior communicating artery (PComA) to the left ICA after occlusion of the left cervical ICA (Figure 4E) and the absence of cross-filling in the right PComA to the left ICA after occlusion of the left cervical ICA (Figure 4F). The patient tolerated a BTO test for the left ICA for 30 mins but failed an additional hypotensive challenge that involved lowering the mean arterial pressure by 20%. Routine frontotemporal craniotomy was performed by cutting along the zygomatic arch, allowing the lateral temporal dura to be opened, and the temporal approach was elected to expose the medial middle fossa and the parasellar and orbital regions of the tumor. The Sylvian fissure was dissected down by retracting the frontal lobe and temporal tip after descending on the optic nerve and tentorial edge. Anterior clinoidectomy was performed with unroofing of the orbital apex. The optic canal and adjacent posterior part of the roof of the orbit were removed with a drill to decompress the optic nerve. The upper and lower dural rings around the paraclinoid ICA were loosened with scissors to expose the cavernous portion of the ICA. The superior wall of the cavernous sinus was incised from the clinoid triangle to the posterior clinoid to adequately expose the cavernous ICA. En bloc resection of the nerve-free tumor segment through the cavernous sinus was performed using an ultrasound dissector, followed by dissection of the tumor from the nerves through the cavernous sinus. Bleeding from the tear in the ICA and its branches was stopped by bipolar cauterization. Radical resection of the extensive recurrent malignant tumor and cavernous and petrous portions of the ICA was achieved with subsequent IMA-RA-MCA bypass (Figures 4G–K). The blood flow within the bypass graft was 61 ml/min, and ICG angiography showed sufficient bypass patency (Figure 4L). All tumors involving bone and the dura mater were extensively removed (Figures 4M–P). The postoperative course was uneventful and followed by adjuvant radiotherapy, and there were no new neurological deficits, except for transient cerebrospinal fluid rhinorrhea, which was resolved through lumbar drainage. Postoperative imaging showed good graft patency of the anastomoses without ischemic changes (Figure 4L). At the 2-year follow-up, the tumor was stable (Figures 4Q,R). There is no signifificant difference in metabolism of left hemisphere between preoperation and the replacement bypass at the three-month follow-up (Figures 4S–V).

## Discussion

### Patient Selection

There are few indications for maximal resection of middle cranial fossa tumors with ICA encasement due to the use of new technologies such as endoscopic surgery, with fewer cases requiring high-flow vessel replacement bypass (11, 12). Maximal resection of tumors with encasement/invasion of the ICA may be more technically challenging not only for middle cranial fossa surgery but also for bypass surgery to revascularize the ICA. The surgical paradigm for radical resection with high functional morbidity has shifted toward promoting tailored resection under neuromonitoring to allow for optimal functional outcomes and then supplemental treatment of suspicious residual lesions by stereotactic radiosurgery and radiotherapy. Although standard fashions such as endonasal approach or open surgery without cerebral revascularization to treat such formidable can achieve satisfactory results (13–16), the majority of patients in our series are treated with unsatisfactory attempt at tailored resection by irradiation with incomplete and ineffective modalities. In our series, the r-knife failed to control the initial residual tumor in 3 patients with recurrence; these patients were followed by subtotal resection of 2 meningiomas and 1 hemangiopericytoma, indicating poor tolerance to new radiation treatment. A second surgical resection was the treatment of choice for 2 patients with adenoid cystic carcinoma and chordoma recurrence, which manifested as decompression and irradiation symptoms. Maximal resection with revascularization yields higher tumor control rates than conservative resection for tumors encasing the ICA, and disappointing results have been found in the supplemental treatment of residual tumors with adjuvant radiotherapy. Five patients in this series had a previous history of conservative resection at another local hospital, 3 of whom subsequently underwent adjuvant r-knife treatment, resulting in short-term tumor recurrence. These patients experienced poor quality of life and a compromised life expectancy due to surgical retreatment and adjuvant radiation. This shift away from incomplete and ineffective treatments suggests that middle cranial fossa tumors referred for open surgery are increasingly complex and time consuming and that neurosurgeons need to be technically proficient in performing combined middle fossa and bypass surgery. Despite the lack of clarity on the indications for treating tumors demonstrating ICA encasement with adjuvant replacement bypass and other various procedures, replacement bypass for these tumors remains an important procedure that can confer modest benefits to patients in the form of maximal tumor resection and saving the patient's brain from ischemic complications *via* intentional or accidental occlusion of the ICA during middle cranial fossa tumor resection. In this series, ICA revascularization was optimal for benign tumors due to the lack of a need for postsurgical local tumor control and radiotherapeutic measures and the absence of craniopharyngioma recurrence complicated by symptomatic fusiform ICA dilation. Carotid artery sacrifice for maximal resection was shown to increase the long-term survival of 2 patients with malignant tumors.

In our cases, the results of the surgery should be better than those of natural tumor progression without bypass surgery. The decision should be made based on the patient's expectations regarding the effect of the surgery on quality of life. Decision-making regarding ICA preservation should still be individualized to determine the costs of subtotal tumor resection vs. ICA resection to achieve maximal tumor resection. IMA bypass, a new method gaining prominence in the field (17), has become an effective method for achieving the radical resection of recurrent craniopharyngioma combined with carotid artery expansion, even if two patients in this report did not require maximal resection of a tumor encasing the ICA.

### Nuances of the Techniques

For resection of middle cranial fossa tumors, craniotomy down to the floor of the middle cranial fossa to the greatest extent possible is preferable and compatible with exposure of the donor IMA in the inferior temporal fossa through frontotemporal craniotomy with resection of the zygomatic arch. IMA-MCA bypass allows for adequate graft flow capacity and revascularization and decreases the potential risk of graft occlusion due to kinking or compression caused by the graft length or tunneling in conventional cervical ICA-MCA or RA bypass (17, 18). BTO may be used to assess patency of the circle of Willis and can be used as a reliable verification of ICA resection without reconstruction or bypass if the occlusion is tolerated. In this series of nine cases, we encountered one case in which sharp dissection of the tumor from the supraclinoid ICA resulted in a tear. The tear was repaired with 8–0 sutures to stop the bleeding by severely narrowing the parent artery. Then, interposition with the STA graft was used as an alternative to suturing the tear by trapping the laceration. Ultimately, this patient developed a severe MCA territory infarction related to ICA and PComA occlusion despite adequate occlusion tolerance exhibited by full collateral blood flow cross-filling from the anterior communicating artery (AComA) on angiography. Appropriate revascularization seemed to benefit patients who underwent ICA resection. Even patients who pass the BTO test may be at risk for serious cerebral infarction. One patient showed compromised severe hemispheric infarction related to occlusion of the PComA flow collateral to the anterior circulation caused by a supraclinoid tear.

Following the confirmation of bypass patency with the new cerebral circulation, the surgeon can carefully dissect the tumor from the involved cranial nerves rather than during the more stressful period when the patient is exhibiting temporary arterial occlusion or a possible complication from vessel invasion by the tumor. In cases of tumor encasement of the ICA, frontotemporal craniotomy can be performed *via* additional osteotomies using the craniozygomatic, cranioorbital or anterior petrosal approach for tumors of the middle fossa extending to the orbital apex, petroclivus or sellar region. For malignant tumors, the basic surgical technique is devascularization of the tumor and en bloc resection with tumor-free margins, including ICA sacrifice to control local disease. There is a potential risk of injury to the contralateral petrous and cavernous ICA when tumor dissection extends beyond the midline to the petroclival area or cavernous medial wall. In our cases, one contralateral ICA injury occurred, which was associated with an unidentified anatomic landmark of chordoma extension due to invasion. Bleeding of the contralateral ICA was fortunately controlled by gluing 1–2 mm lengths of muscle. For benign tumors, the clinoid and oculomotor spaces were used for intracavernous tumors, while for tumors that extended to the prepetrous and dorsal sellar regions, additional space was created between the oculomotor and trochlear nerves through the prepetrous approach. Anterior clinoidectomy is a key access procedure and an important step for ICA siphoning through the region between the oculomotor and clinoid spaces. The distal ICA of the intracavernous sinus is exposed by scissoring the upper and lower dural rings around the paraclinoid ICA. Removal of the petrous apex and dorsum sellae also allows access to the petrous ascending segment of the intracavernous ICA and is suitable for the resection of all intracavernous tumors, including intracavernous dura removal. For the dissection of intracavernous tumors through the anterior clinoid and oculomotor spaces, the anterior clinoid processes serve as landmarks of the nerves through the intracavernous sinus because they remain relatively consistent. The optic nerve is identified on the medial side of the clinoid processes; the oculomotor nerve is on the lower side of the clinoid processes (19). These cranial nerves in the intra/lateral sinus wall are susceptible to damage due to tumor engulfment. After the cranial nerves adherent to the tumor in the cavernous sinus are carefully dissected, an ultrasound dissector or powerful suction can be applied to ease removal of these tumors.

### Report Limitations

Long-term radiographic patency and clinical follow-up examinations of patients treated with IMA bypass for middle fossa tumors should be required. This report has a few limitations, including its retrospective design and patient selection bias; additionally, middle cranial fossa tumors encasing/invading the ICA are rare entities, and this study included only 9 cases.

## Conclusions

Maximal surgical resection of middle cranial fossa tumors with ICA encasement or invasion is possible through revascularization, as higher flow IMA-MCA bypass with a shorter radial graft in a single microsurgical field is comparable to craniotomy of the middle cranial fossa. The salvage surgical resection of tumors still requires a rather eventful course of critical postoperative management for cerebral infarction, CSF leakage, and hydrocephalus, leading to a dismal prognosis for patients with malignant tumors and resulting in unnecessary and incomplete retreatments later after tumor recurrence.

## Data Availability Statement

The raw data supporting the conclusions of this article will be made available by the authors, without undue reservation.

## Author Contributions

YS, LW, and XS: conception and design. YS and LW: drafting of the article. LW and XS: critical revision of the article and study supervision. All authors: acquisition of data. All authors contributed to the article and approved the submitted version.

## Funding

This work was supported by the Beijing Municipal Natural Science Foundation (Grant No. 7161005 to XS) and the Science and Technology Commission Foundation of Beijing (Grant No. Z161100000516019 to XS).

## Conflict of Interest

The authors declare that the research was conducted in the absence of any commercial or financial relationships that could be construed as a potential conflict of interest.

## Publisher's Note

All claims expressed in this article are solely those of the authors and do not necessarily represent those of their affiliated organizations, or those of the publisher, the editors and the reviewers. Any product that may be evaluated in this article, or claim that may be made by its manufacturer, is not guaranteed or endorsed by the publisher.
